# Drug response prediction model using a hierarchical structural component modeling method

**DOI:** 10.1186/s12859-018-2270-7

**Published:** 2018-08-13

**Authors:** Sungtae Kim, Sungkyoung Choi, Jung-Hwan Yoon, Youngsoo Kim, Seungyeoun Lee, Taesung Park

**Affiliations:** 1 Interdisciplinary Program in Bioinformatics, Seoul National University, Seoul, 08826 South Korea; 2 Department of Internal Medicine and Liver Research Institute, Seoul National University College of Medicine, Seoul, 03080 South Korea; 3 Department of Biomedical Engineering, Seoul National University College of Medicine, Seoul, 03080 South Korea; 4 Department of Mathematics and Statistics, Sejong University, Seoul, 05006 South Korea; 5 Department of Statistics, Seoul National University, Seoul, 08826 South Korea

**Keywords:** Biomarkers, Component-based structural equation modeling, Drug response, Liver cancer, Multiple reaction monitoring mass spectrometry (MRM-MS), Prediction model, Sorafenib

## Abstract

**Background:**

Component-based structural equation modeling methods are now widely used in science, business, education, and other fields. This method uses unobservable variables, i.e., “latent” variables, and structural equation model relationships between observable variables. Here, we applied this structural equation modeling method to biologically structured data. To identify candidate drug-response biomarkers, we first used proteomic peptide-level data, as measured by multiple reaction monitoring mass spectrometry (MRM-MS), for liver cancer patients. MRM-MS is a highly sensitive and selective method for proteomic targeted quantitation of peptide abundances in complex biological samples.

**Results:**

We developed a component-based drug response prediction model, having the advantage that it first combines collapsed peptide-level data into protein-level information, facilitating subsequent biological interpretation. Our model also uses an alternating least squares algorithm, to efficiently estimate both coefficients of peptides and proteins. This approach also considers correlations between variables, without constraint, by a multiple testing problem. Using estimated peptide and protein coefficients, we selected significant protein biomarkers by permutation testing, resulting in our model for predicting liver cancer response to the tyrosine kinase inhibitor sorafenib.

**Conclusions:**

Using data from a cohort of liver cancer patients, we then “fine-tuned” our model to successfully predict drug responses, as demonstrated by a high area under the curve (AUC) score. Such drug response prediction models may eventually find clinical translation in identifying individual patients likely to respond to specific therapies.

## Background

Liver cancer (hepatic cancer), is predominantly found in the tissue parenchyma, and is thus known as hepatocellular carcinoma (HCC), the most common form of liver cancer in adults. HCC can exert different growth patterns from one tumor to the next [1, 2]. In Eastern Asia, HCC is the third-most common form of cancer, and the second-leading cause of cancer death, with a worldwide total of 600,000 deaths each year [3, 4]. However, as many treatment methods have been developed for treating HCC, overall, these have shown little benefit in improving patients’ prognosis [5]. More efficient treatment of HCC may lie in “personalized medicine,” i.e., tailoring therapies for individual patients [6]. Such ability to classify HCC patients, with therapies optimized for specific stage and growth patterns, would reduce time and cost, and likely prolong survival.

Toward that objective, accurate prediction models are essential. Historically, methods of building cancer prediction models were based on the classification methods of linear/logistic regression, support vector machine, or random forest [7–9]. While these models are effective for prediction, they do not consider any structural or hidden biological data, making it difficult to derive more meaningful biological interpretation.

Here, we built a drug response prediction model, by identifying candidate protein biomarkers, via multiple reaction monitoring-mass spectrometry (MRM-MS) technology. MRM-MS is a targeted proteomics technology that is highly selective and sensitive for quantitating targeted proteins or peptides in biological samples [10, 11]. MRM-MS can also measure several hundred protein targets per sample, simultaneously, generating consistent, precise, and reproducible datasets [12]. Consequently, MRM-MS holds high potential for biomarker discovery. Unlike other protein data, MRM-MS data is hierarchically structured.

Following mass spectrometry, our MRM-MS data consisted of 231 peptides, from 124 proteins, with each protein containing ≥1 peptide. While classical methods for prediction model building only select the best peptides as variables, to optimize prediction performance, these do not consider any biological relationship between peptides and proteins.

In this study, we built a drug response prediction model, using a component-based structural equation modeling method, based on the biological structure of MRM-MS data (e.g., peptide to protein). Structural equation modeling (SEM) is used to analyze the structural relationship between unobserved (latent) variables and observed variables. SEM can be classified as factor based SEM and component based SEM. Confirmatory factor analysis (CFA) and partial least squares path modeling (PLS-PM) analysis are the most popular methods of factor based SEM and component based SEM, respectively [13]. Our proposed model is based on generalized structured component analysis (GSCA) [14], resembling our earlier derivation of a pathway-based approach. That analysis, using hierarchical components of collapsed rare variants (PHARAOH), uses a hierarchical structure of pathways and genes [15].

Using latent variables, we can collapse multiple peptides into a structured form of proteins that they comprise of, providing more feasible biological explanations of the results. In addition to hierarchical structure, we further showed that HisCoM effectively cover protein-level analysis, taking all peptides into account simultaneously. Moreover, for real biological data analysis, using MRM-MS, we discovered possible protein biomarkers associated with patients’ response to the multiple tyrosine kinase inhibitor sorafenib (Nexavar®) [16]. Sorafenib is known as effective and safe drug for recovering liver cancer (hepatocellular carcinoma) patients not only Asian-Pacific region but also in the North American region [17, 18]. Using these protein biomarkers, we then evaluated the performance of our drug response prediction model. Additionally, we compared the performance of our prediction model, using area under the curve (AUC) scores, to performances by generalized linear models of logistic regression, without ridge parameters, and logistic regression, with ridge parameters. Furthermore, through extensive simulation studies, we compared the performance of our proposed method with other logistic regression methods. For hierarchical structuring, in this case, for proteins with multiple peptides, our HisCoM was shown to perform better than logistic regression, as assessed by AUC scores.

## Methods

### Preparing samples and materials

Hepatocellular carcinoma (HCC) patient serum samples (*n* = 115) were collected at Seoul National University Hospital, from 2013 to 2015 [19]. Upon diagnosis of liver cancer, patients were placed on a regimen using the tyrosine kinase inhibitor Sorafenib (Nexavar®, Bayer, Inc., Whippany, NJ, USA). Patients’ tumor sizes were first examined immediately following HCC diagnosis, at the start of hospital admission. Six weeks after first diagnosis (sufficient time to see a response), patients’ tumors were again measured, by contrast-enhanced computed tomography or magnetic resonance imaging, and staged according to the standardized Modified Response Evaluation Criteria in Solid Tumors (mRECIST) [20]. After the second examination, patients were divided into two groups, based on positive and negative drug responses. The positive drug response group consisted of patients with complete response (CR), partial response (PR), or stable disease (SD), according to mRECIST [20]. CR and PR responses were diagnosed when the tumor size was reduced after 6 weeks. Also, SD was diagnosed when the size of the tumor remained stable, from the first to second visit. On the other hand, the negative drug response group consisted of patients with progressive disease (PD), wherein the size of their tumors increased, from first diagnosis to 6 weeks later. The study protocol was approved by the Institutional Review Board of Seoul National University Hospital (IRB No. 0506–150-005), and written, informed consent was obtained from each patient or legally authorized representative.

Among all 115 patients (101 men and 14 women), 40 patients (37 men and 3 women) were grouped into the positive drug response group, and 75 (64 men and 11 women) were grouped into the negative drug response group. From each patient’s serum, data for 231 peptides was generated by multiple reaction monitoring mass spectrometry (MRM-MS), a highly sensitive and selective method for targeted quantitation of peptide abundances, in complex biological samples [21]. Here, the 231 peptides can represent 124 proteins. Since the MRM-MS technique measures the quantity of targeted peptides in patients’ serum, we used the log2-transformed ratio of light peptide intensity to heavy peptide intensity. Light peptide intensity represented the quantity of peptides from specific patient’s blood, as measured by MRM-MS, while heavy peptide intensity represented the quantity of artificially built, same sequences as the light peptides, but using heavier isotope elements, also as measured by MRM-MS. The software Skyline was used to measure the intensity of light and heavy peptides by MRM-MS [22]. Demographic information, such as age and sex, were also available. The range of age varied from 34 to 84, with 101 male and 14 female samples.

### Constructing the drug response model

**Fig. 1:**
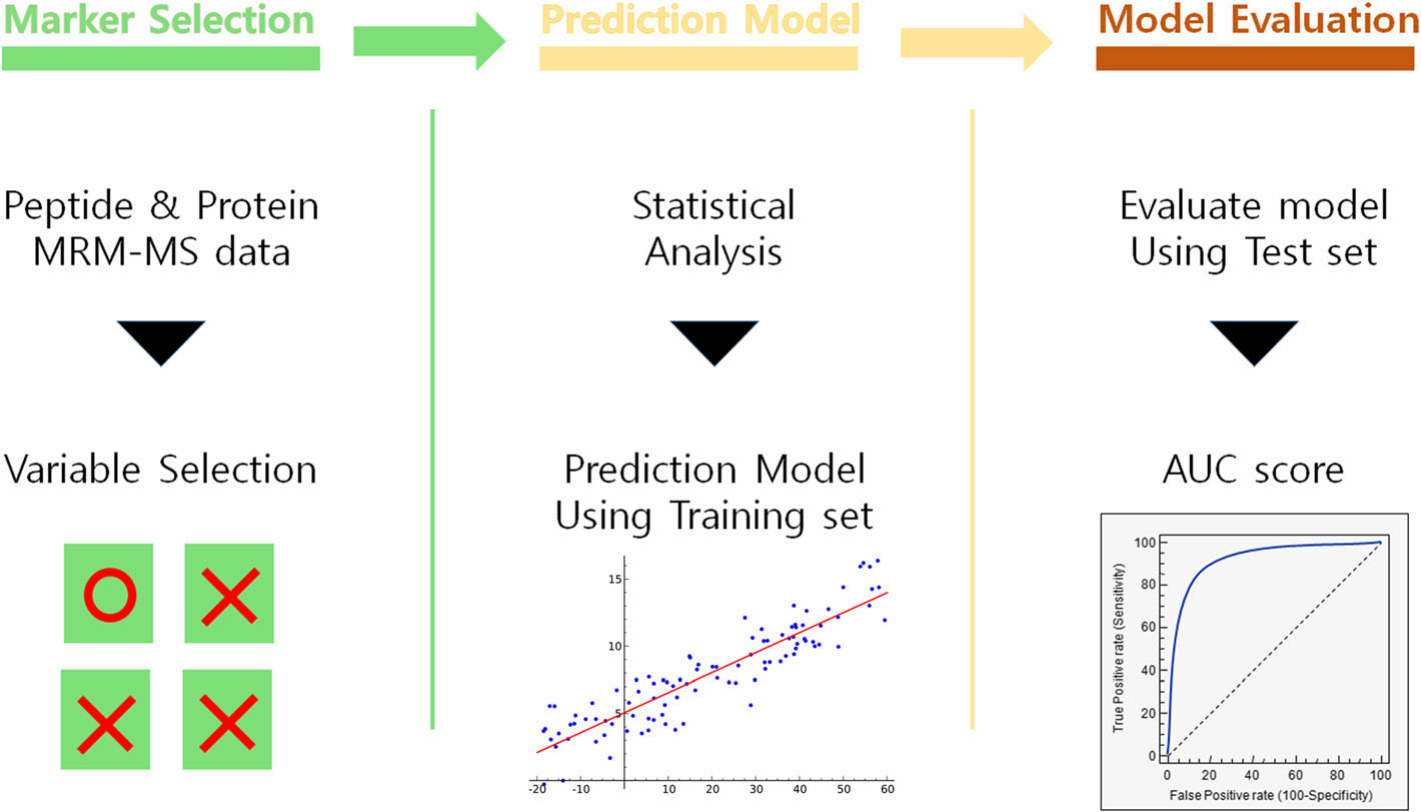
Schematic procedure of overall analysis

The overall schematic procedure is shown in Fig. 1. At the beginning, we selected protein level biomarkers by HisCoM method with 1000 permutation test, for possible prediction of sorafenib response, using MRM-MS data. Second, we constructed prediction models, via a component-based structural equation-modeling method. Finally, we evaluated the constructed drug response prediction models’ performances, by AUC scores.

**Fig. 2:**
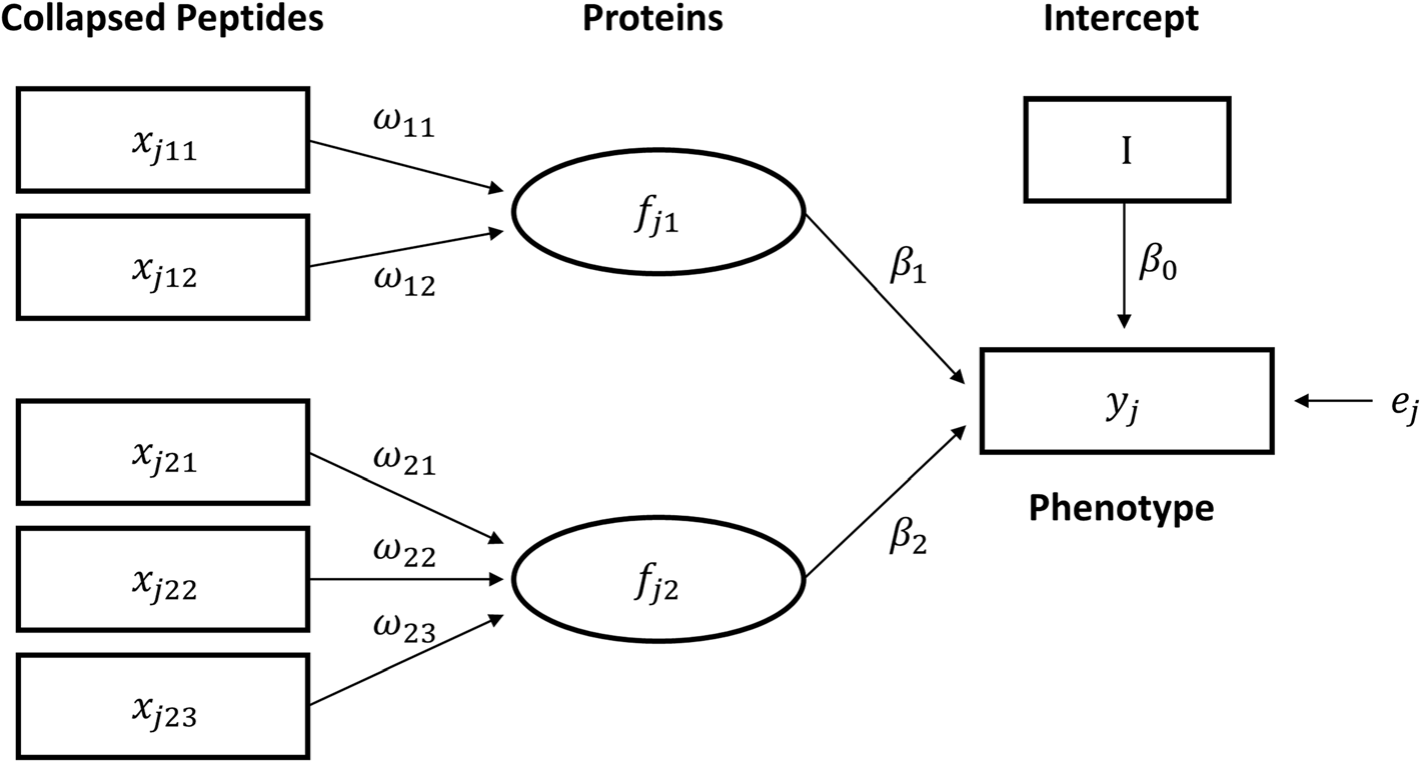
Example of proposed component-based structured equation model

An example of our proposed drug response prediction model is shown in Fig. 2. This model combines collapsed peptide-level MRM-MS data into protein-level information, and efficiently estimates both peptide and protein coefficients. In this example, two proteins were involved (*K* = 2), and each protein consisted of two or three peptides (T_*k*_ = 2 and 3). Weight (*w*) and path coefficients (*β*) were estimated using alternating least squares [23].

Here, suppose that there are *K* proteins, and the *k*^th^ protein contains T_*k*_ peptides, for *k* = 1,…,*K*. To estimate parameters, the following penalized log likelihood function was maximized. The *y*_*j*_ represent the drug response group, based on mRECIST: *y*_*j*_ = 0 for a negative response, and *y*_*j*_ = 1 for a positive response. Let *y*_*j*_ is distributed independently with a mean of *μ*_*j*_ = E[*y*_*j*_] and *η*_*j*_ is defined as *η*_*j*_ = *g*(*μ*_*j*_) by a logit link function *g*. Then, we can derive a penalized log likelihood function with dispersion parameter *δ* and canonical parameter *γ*_*i*_ as following:

1 $$ {\varphi}_1=\sum \limits_{j=1}^N\log P\left({y}_j;{\gamma}_i,\delta \right)-\frac{1}{2}{\lambda}_{pep}\sum \limits_{k=1}^K\sum \limits_{t=1}^{T_k}{w}_{kt}^2-\frac{1}{2}{\lambda}_{prot}\sum \limits_{k=0}^K{\beta}_k^2 $$

Here, *λ*_*prot*_ and *λ*_*pep*_ are the ridge parameters for proteins and peptides represent as “tuning” parameters, respectively: one for the peptides within a protein and the other for the proteins themselves.

Let ***w***_*k*_ = [*w*_*k*1_*,⋯,*${w}_{k{T}_k}$], ***β*** = [*β*_0*,*_ *β*_1_*,⋯, β*_*K*_], ***F*** = [*f*_1_*,⋯, f*_*N*_], and ***f***_*j*_ = [1, *f*_*j*1_*,⋯, f*_*jK*_] where ${f}_{jk}=\sum \limits_{t=1}^{T_k}{x}_{jk i}{w}_{ki}$. We define *x*_*jki*_as the quantity of *i*^*th*^ peptide of the *k*^*th*^ protein in sample *j*. The *w*_*ki*_ as a weight coefficient of *i*^*th*^ peptide of the *k*^*th*^ protein. Also, the *β*_*k*_ as a path coefficient of *k*^*th*^ protein. Maximizing the eq. (1), via iteratively reweighted least squares, is identical to minimizing the following penalized least squares eq. (2):

2 $$ {\displaystyle \begin{array}{c}{\varphi}_2=\sum \limits_{j=1}^N{v}_j{\left({z}_j-\sum \limits_{k=0}^K{f}_{jk}{\beta}_k\right)}^2+{\lambda}_{pep}\sum \limits_{k=1}^K\sum \limits_{t=1}^{T_k}{w}_{kt}^2+{\lambda}_{prot}\sum \limits_{k=0}^K{\beta}_k^2\\ {}={\left(\boldsymbol{z}-\boldsymbol{F}\boldsymbol{\beta } \right)}^{\prime}\boldsymbol{V}\left(\boldsymbol{z}-\boldsymbol{F}\boldsymbol{\beta } \right)+{\lambda}_{pep}\sum \limits_{k=1}^K\left({{\boldsymbol{w}}_k}^{\prime }{\boldsymbol{w}}_k\right)+{\lambda}_{prot}\left({\boldsymbol{\beta}}^{\prime}\boldsymbol{\beta} \right)\end{array}} $$

with respect to ***w***_*k*_ and ***β*** [15, 24]. Here, ***V*** is an *N* by *N* diagonal matrix with elements ${v}_j=\frac{{\left({\partial}_{\mu_j}/{\partial}_{\eta_j}\right)}^2}{\tau_j}$. *τ*_*j*_ is the variance function evaluated at *μ*_*j*_. The ***z*** is the adjusted response variable and an *N* × 1 vector with elements ${z}_j={\eta}_j+\frac{\left({y}_j-{\mu}_j\right)}{v_j}$ [25].

After estimating the *w*_*ki*_ and *β*_*k*_ coefficients, we constructed a drug response prediction model for *π*_*j*_ = *P*[*y*_*j*_ = 1] = *μ*_*j*_, as follows, after standardization of *x*_*jki*_ The coefficients of age and sex are also estimated by maximizing the log-likelihood function simultaneously while penalizing the coefficients of peptides. In our final prediction model, the beta coefficients of age and sex are fixed across the individuals.

3 $$ \mathit{\log}\left(\frac{\pi_j}{1-{\pi}_j}\right)={\beta}_0+\sum \limits_k\left(\sum \limits_i{x}_{jki}{w}_{ki}\right){\beta}_k+{AGE}_j{\beta}_{age}+{SEX}_j{\beta}_{sex} $$

4 $$ \kern1.5em ={\beta}_0+\sum \limits_k{f}_{jk}{\beta}_k+{AG\mathrm{E}}_j{\beta}_{age}+{SEX}_j{\beta}_{sex} $$

*j*: individual samples (*j* = 1, ⋯,115)

*k*: proteins (*k* = 1, ⋯,124)

*i*: peptides (*i* = 1, ⋯,231)

**Fig. 3:**
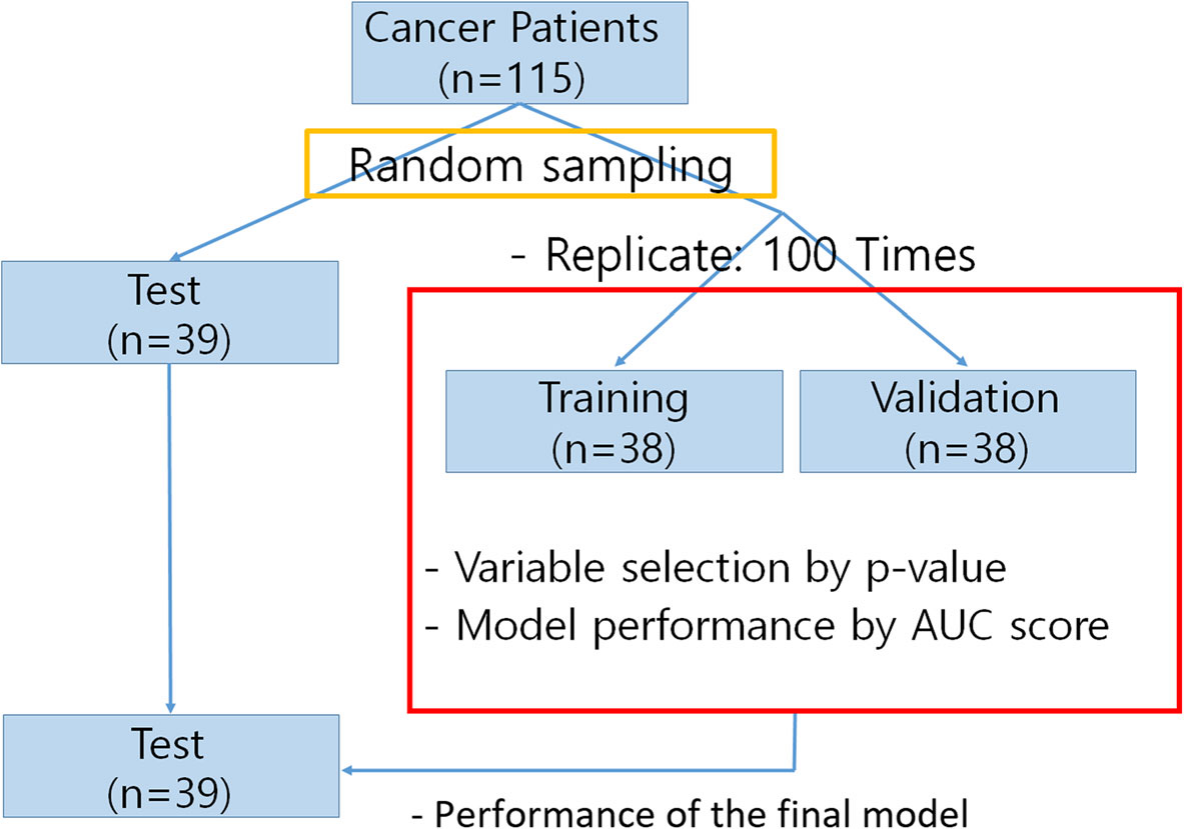
Sample separation for training, validation, and test sets

When our final drug response prediction model was constructed, we evaluated its performance by area under the receiver operating characteristic curve (AUC) score, based on the training, validation, and test sets. The approach for separating the training set from the validation and test sets, is depicted in Fig. 3. First, we then randomly selected 39 out of 115 samples (35 men and 4 women) as a test set, excluded from the modelling process, while assessing the remaining 76 samples. This test set will be used to measure the performance of our final drug response prediction model. The ratio of positive responses to negative responses was sustained (14 positive responses and 25 negative responses). The range of age in test set was distributed from 41 to 84. The remaining 76 samples were randomly (without replacement) divided into training and validation set. For a fair comparison, the ratio of positive responses to negative responses was retained (13 positive responses and 25 negative responses). The concept of a sample separation process was based on a previously developed intraductal papillary mucinous neoplasm (IPMN) patient prediction model [26].

Lastly, our drug response prediction model was compared to the generalized linear model with a binary response (GLM), and the generalized linear regression with a binary response via ridge parameter (GLMwR) methods. All the analyzes were calculated and computed via software R (Version R 3.2.3) [27].

### Simulation design

For the simulation study, we designed two models: the first model composed of two significant proteins and the second model with both significant and nonsignificant protein in the presence of a hierarchical structure of MRM-MS data (e.g., peptide to protein). Let the first simulation model contain JCHAIN and RBP4 with parameters estimated by HisCoM. Note that JCHAIN was a significant protein (*p*-value: 0.0142), with 3 peptides, and RBP4 was also a significant protein (*p*-value: 0.0031), with 2 peptides. The simulation model is given by

5 $$ {\displaystyle \begin{array}{l}\log \left(\frac{\pi_j}{1-{\pi}_j}\right)={\beta}_0+\left(\sum \limits_{i=1}^3{x}_{ji}{\omega}_i\right){\beta}_{IGJ}+\left(\sum \limits_{i=4}^5{x}_{ji}{\omega}_i\right){\beta}_{RET4}\\ {}+{AGE}_j{\beta}_{age}+{SEX}_j{\beta}_{sex}\end{array}} $$

For the Simulation model 2, we assume the true model contains RBP4 and APOA1, with parameters estimated by HisCoM. Note that RBP4 was a significant protein (*p*-value: 0.0031), with 2 peptides, and APOA1 was a nonsignificant protein (*p*-value: 0.4794), with 7 peptides. The second simulation model is given by

6 $$ {\displaystyle \begin{array}{l}\log \left(\frac{\pi_j}{1-{\pi}_j}\right)={\beta}_0+\left(\sum \limits_{i=1}^2{x}_{ji}{\omega}_i\right){\beta}_{RET4}+\left(\sum \limits_{i=3}^9{x}_{ji}{\omega}_i\right){\beta}_{APOA1}\\ {}+{AGE}_j{\beta}_{age}+{SEX}_j{\beta}_{sex}\end{array}} $$

In this case, *x*_*j*, 1_ represents the *j*^*th*^ individual’s peptide data (*x*_*j*,1_, *x*_*j*,2_, ⋯, *x*_*j*,9_). From the estimated *β*s and *ω*s, derived from the data, we estimated *π*_1_, *π*_2_,⋯, *π*_115_. Then, the responses were generated from the Bernoulli distribution B(1, *π*_*j*_), for *j* = 1, 2, ⋯, 115. We then constructed HisCoM, GLM, and GLMwR drug response prediction models, using MRM-MS peptide data (*x*_*j*,1_, *x*_*j*,2_,⋯, *x*_*j*, 9_), to generate response variables. For each simulation model, we measured the AUC score. Using the same estimated values of *π*_1_, *π*_2_,⋯, *π*_115_, we repeated the whole process 1000 times, and obtained 1000 AUC scores for each of the HisCoM, GLM, and GLMwR models. We then calculated the mean of the 1000 AUC scores, based on those models.

## Results

### Biomarker discovery for the drug response prediction model

To evaluate our model, at the beginning, we randomly selected 39 out of 115 samples, as a separate, test set, to evaluate the overall performance of the final drug response prediction model. We performed cross-validation analysis using remaining 76 samples. The dataset was randomly divided into training/validation sets (38 samples for each set). From the training data set, the significant proteins were selected based on *p-*values. Then the prediction model was build, and its AUC score was computed from the validation set. We repeated this cross-validation 100 times. Through 100 cross-validation, we evaluated whether the significant proteins were selected repeatedly by HisCoM. Also, using the estimated path coefficients by training set, we evaluated the performance of the prediction model from the validation set.

The significances of the protein path coefficients were then determined, using a 1000 permutation test, for each replicate. The permutation test was performed by shuffling drug response variables randomly across subjects while retaining the ratio of positive response to negative response and then estimating the path coefficients. Using path coefficients estimated by 1000 permutation test, we construct distribution of each protein’s path coefficient. Through comparing the path coefficient value of the original data with those from the permuted data, *p*-values were computed for each protein, with the significant (*P* < 0.05) ones being selected for further analysis.

**Fig. 4:**
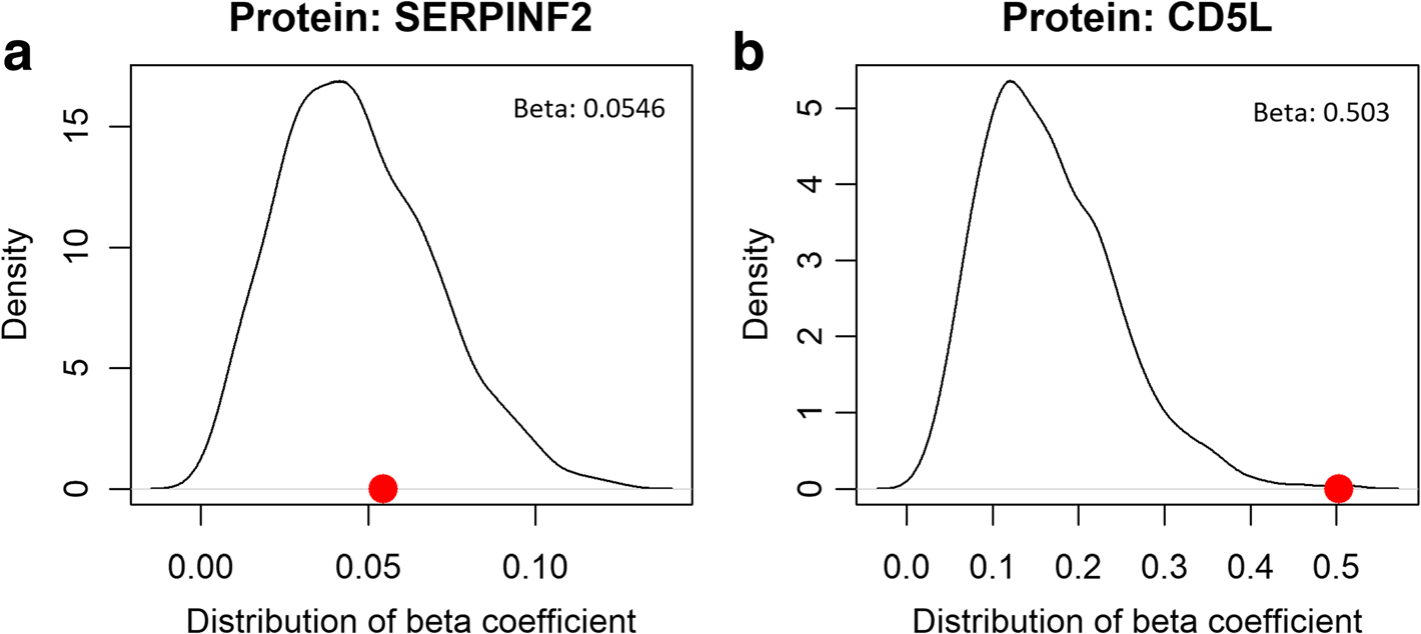
Estimation of beta coefficients (path coefficients) for each protein example. **a** non-significant proteins; **b** significant proteins. Red dot indicates the estimated path coefficient of the protein

Figure 4 shows the null distribution of path coefficients (*β*_*k*_), derived from 1000 permutation tests. Figure 4a show the case of non-significant protein, while the Fig. 4b does the case of significant protein. The red dots indicate the estimated path coefficients from the data. In our analysis, the path coefficients (*β*_*k*_) of six proteins were significant.

During the processing of the training/validation sets, with 76 samples, we repeated this process 100 times, to check the consistency of possibly significant proteins. Since our method uses two ridge parameters, we defined the same tuning parameter values as 10 for peptides and proteins, for computational efficiency. As a result, we selected the top 6 significant proteins (APOC4, CD163, CD5L, JCHAIN, SERPING1, and RBP4), which were repeatedly selected as significant by the process of 100 replications. We noted that these six proteins were previously identified as possible proteomic biomarkers, for hepatocellular carcinoma [28–30].

We then repeated the process once more, with only those 6 proteins, as MRM-MS data, for more accurate estimation of *p-*values and path coefficients, for the drug response prediction model. We next calculated *p*-values and path coefficients. In Table 1, *p-*values are shown for the six selected proteins.

**Table 1:** *P-*values for our 6 candidate biomarkers: APOC4, CD163, CD5L, JCHAIN, SERPING1, and RBP4, based on MRM-MS data

Protein	*P*-value
APOC4	0.0061
CD163	0.0112
CD5L	0.0031
SERPING1	0.0102
JCHAIN	0.0142
RBP4	0.0031

Using the selected 6 proteins, we constructed a drug response prediction model, with estimated *w* and *β* values. We also constructed different prediction models, limiting the number of proteins. In this case, we constructed models using 1 of the 6 proteins, 2 of the 6, 3 of the 6, and all six. For all these models, age and sex were considered as covariates (see eqs. 3 and 4, below). All the analyzes were calculated and computed via software R (Version R 3.2.3) [27].

### Model evaluation by AUC results

With the selected proteins, we first constructed a sorafenib drug response prediction model given in eq. (4), using HisCoM. The performance of the drug response prediction models was measured by AUC scores. In this case, the numbers *k* and *i* varied, depending on the number of proteins in the model.

**Fig. 5:**
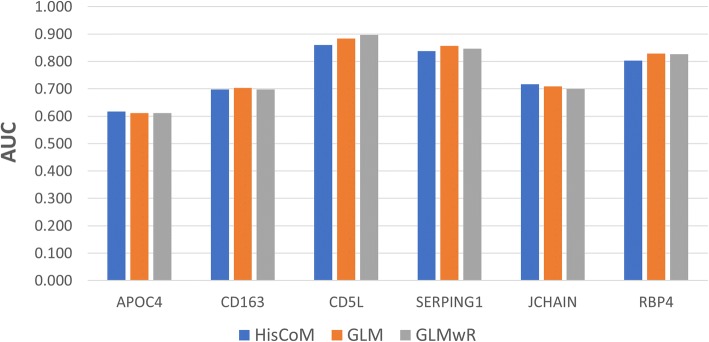
AUC score comparison between HisCoM, GLM, and GLMwR drug response prediction models using single candidate protein

Table 2 shows the AUC score of our single protein prediction model, compared to a corresponding the generalized linear model with a binary response (GLM), and the generalized linear model with a binary response via ridge parameters (GLMwR). The performance of the single protein prediction models showed similar AUC scores, across all three different statistical methods, while the AUC scores, for each individual protein, varied from 0.60 to 0.90. Figure 5 provides a visual comparison of AUC scores, by different single protein statistical models.

**Table 2:** AUC score comparison between HisCoM, GLM, and GLMwR drug response models using single candidate protein

Protein	HisCoM	GLM	GLMwR
APOC4	0.617	0.611	0.611
CD163	0.697	0.703	0.697
CD5L	0.860	0.883	0.897
SERPING1	0.837	0.857	0.846
JCHAIN	0.717	0.709	0.700
RBP4	0.803	0.829	0.826

**Fig. 6:**
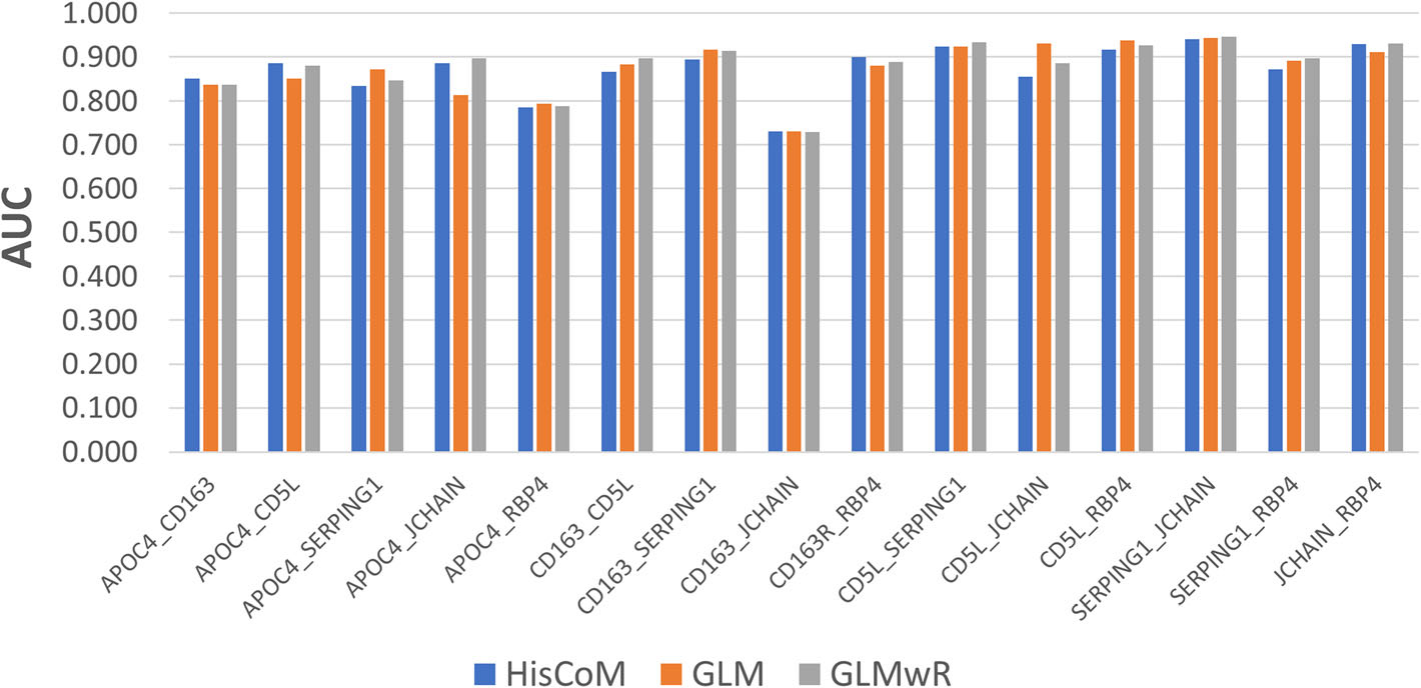
AUC score comparison between HisCoM, GLM, and GLMwR drug response prediction models using double candidate proteins

The prediction model, using 2 of the 6 proteins, had higher AUC scores, compared to the single protein models, across all three different statistical methods. Table 3 shows the AUC scores for the models with 2 of the 6 proteins. The AUC scores across each statistical model varied from 0.73 to 0.95, higher than those for the single protein prediction models. Figure 6 shows a visual comparison, of AUC scores, by different two-protein statistical models. The HisCoM’s AUC score was similar to those of GLM and GLMwR, but had higher performance or lower performance, depending on the combination of proteins. The best performing protein combination, across each HisCoM, GLM, and GLMwR statistical model, was the combination of SERPING1 and JCHAIN.

**Table 3:** AUC score comparison between HisCoM, GLM, and GLMwR drug response models using double candidate proteins

Protein	HisCoM	GLM	GLMwR
APOC4_CD163	0.851	0.837	0.837
APOC4_CD5L	0.886	0.851	0.880
APOC4_SERPING1	0.834	0.871	0.846
APOC4_JCHAIN	0.886	0.814	0.897
APOC4_RBP4	0.786	0.794	0.789
CD163_CD5L	0.866	0.883	0.897
CD163_SERPING1	0.894	0.917	0.914
CD163_JCHAIN	0.731	0.731	0.729
CD163R_RBP4	0.900	0.880	0.889
CD5L_SERPING1	0.923	0.923	0.934
CD5L_JCHAIN	0.854	0.931	0.886
CD5L_RBP4	0.917	0.937	0.926
SERPING1_JCHAIN	0.940	0.943	0.946
SERPING1_RBP4	0.871	0.891	0.897
JCHAIN_RBP4	0.929	0.911	0.931

**Fig. 7:**
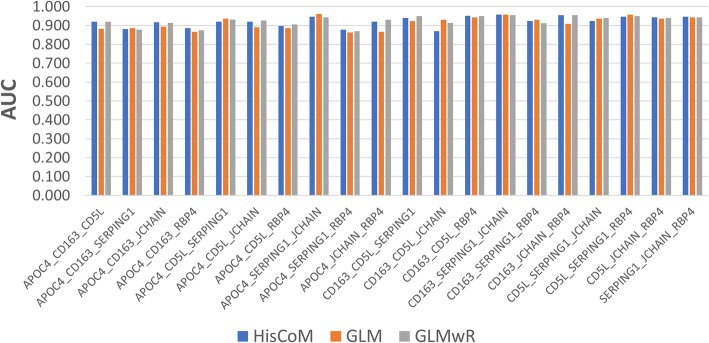
AUC score comparison between HisCoM, GLM, and GLMwR drug response prediction models using triple candidate proteins

Similarly, most of the prediction models, with 3 of the 6 proteins, scored higher than 0.9 AUC, using all three modeling methods. Table 4 shows the AUC scores for each model using exhaustive combinations of the three proteins, varying from 0.86 to 0.95. Figure 7 shows a visual comparison of AUC scores, by different 3-protein statistical models.

**Table 4:** AUC score comparison between HisCoM, GLM, and GLMwR drug response models using triple candidate proteins

Protein	HisCoM	GLM	GLMwR
APOC4_CD163_CD5L	0.920	0.883	0.920
APOC4_CD163_SERPING1	0.880	0.886	0.877
APOC4_CD163_JCHAIN	0.917	0.894	0.914
APOC4_CD163_RBP4	0.886	0.866	0.874
APOC4_CD5L_SERPING1	0.920	0.937	0.931
APOC4_CD5L_JCHAIN	0.920	0.891	0.926
APOC4_CD5L_RBP4	0.897	0.886	0.906
APOC4_SERPING1_JCHAIN	0.946	0.960	0.943
APOC4_SERPING1_RBP4	0.877	0.863	0.869
APOC4_JCHAIN_RBP4	0.920	0.866	0.929
CD163_CD5L_SERPING1	0.940	0.923	0.949
CD163_CD5L_JCHAIN	0.869	0.929	0.914
CD163_CD5L_RBP4	0.951	0.943	0.949
CD163_SERPING1_JCHAIN	0.957	0.957	0.954
CD163_SERPING1_RBP4	0.923	0.929	0.911
CD163_JCHAIN_RBP4	0.954	0.909	0.954
CD5L_SERPING1_JCHAIN	0.923	0.937	0.940
CD5L_SERPING1_RBP4	0.946	0.957	0.949
CD5L_JCHAIN_RBP4	0.943	0.937	0.940
SERPING1_JCHAIN_RBP4	0.946	0.943	0.943

**Fig. 8:**
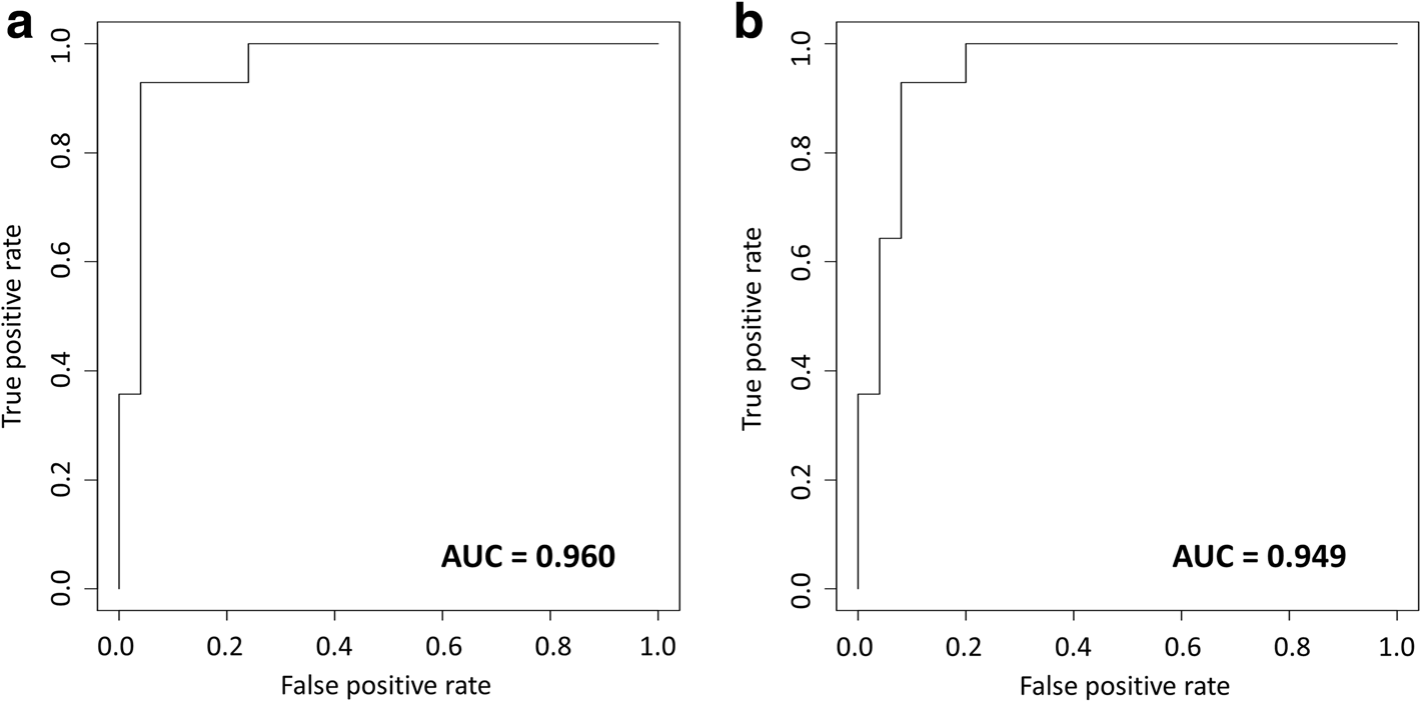
AUC score, using all 6 proteins, for each model. **a** HisCoM. **b** Generalized linear model with ridge parameter

**Fig. 9:**
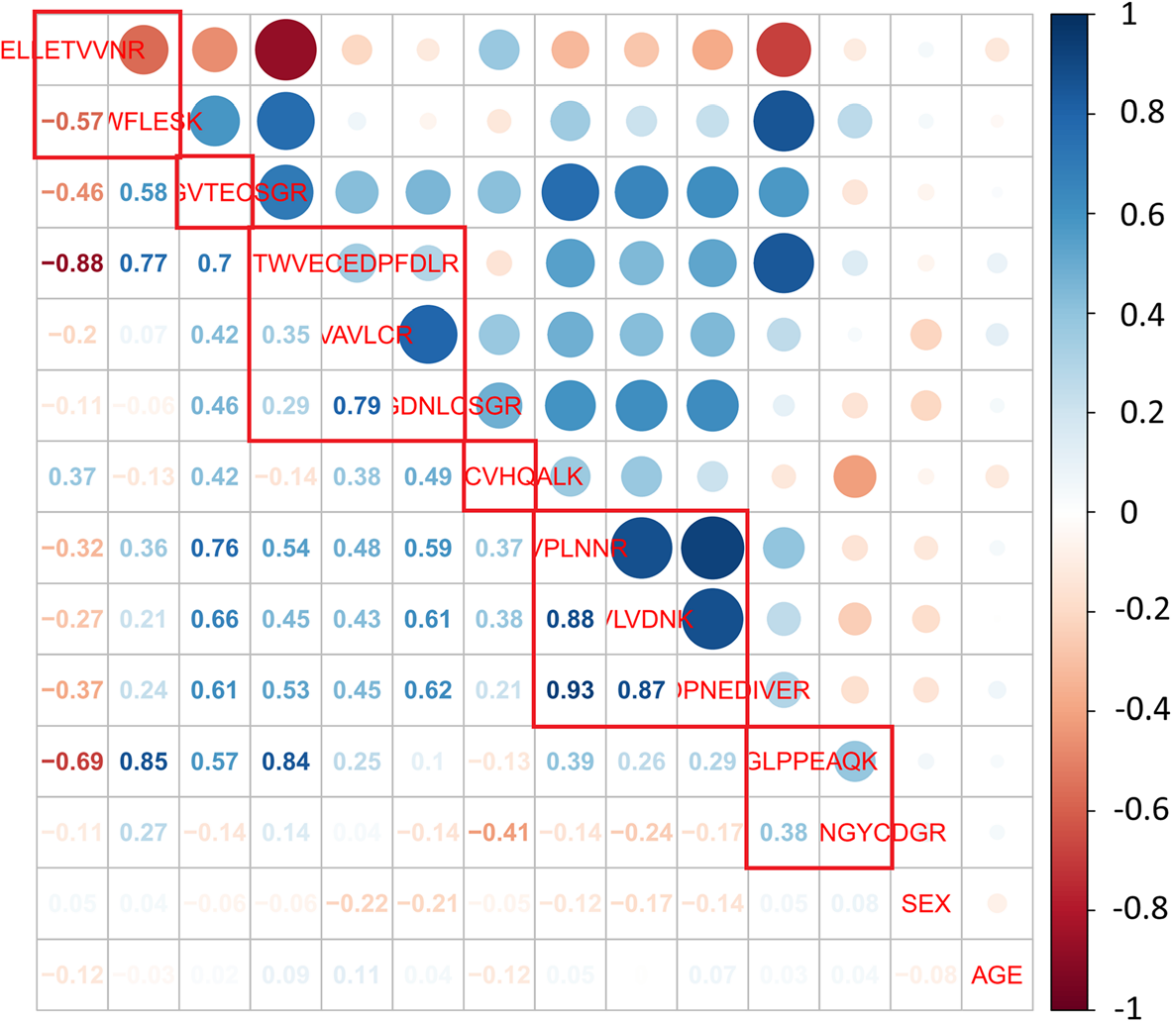
Correlations between peptides. Each red square box represents the peptides within the same protein

Using all 6 proteins, we also constructed HisCoM drug response prediction models, with estimated *ω* and *β* as the covariates age and sex, respectively. In Fig. 8, the AUC score for our HisCoM model was 0.96, using the validation set. At first, we tried to compare our prediction model to the generalized linear model with a binary response. However, the latter had a convergence problem, due to high correlation among peptides, as shown in Fig. 9. To resolve this problem, we fit the logistic regression model with a ridge penalty, using the “GLMNET” R Package. The result is shown in Fig. 8, and the AUC score for the generalized linear model with a binary response via ridge parameter (GLMwR), was 0.949, using the same validation set. As a result, our HisCoM had a slightly better AUC score (0.96), compared to that of GLMwR (0.949).

### Simulation results

**Fig. 10:**
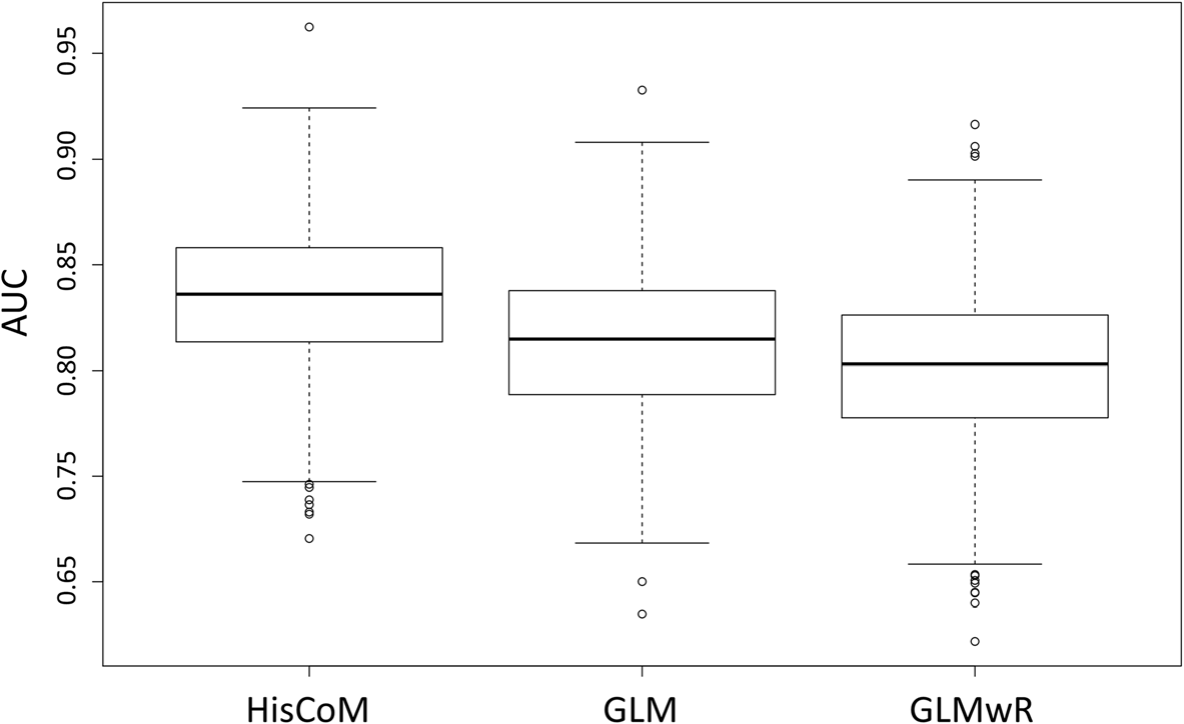
Box plots of ranges of 1000 AUC scores of HisCoM-based simulation data, compared to other models: Simulation model 1

**Fig. 11:**
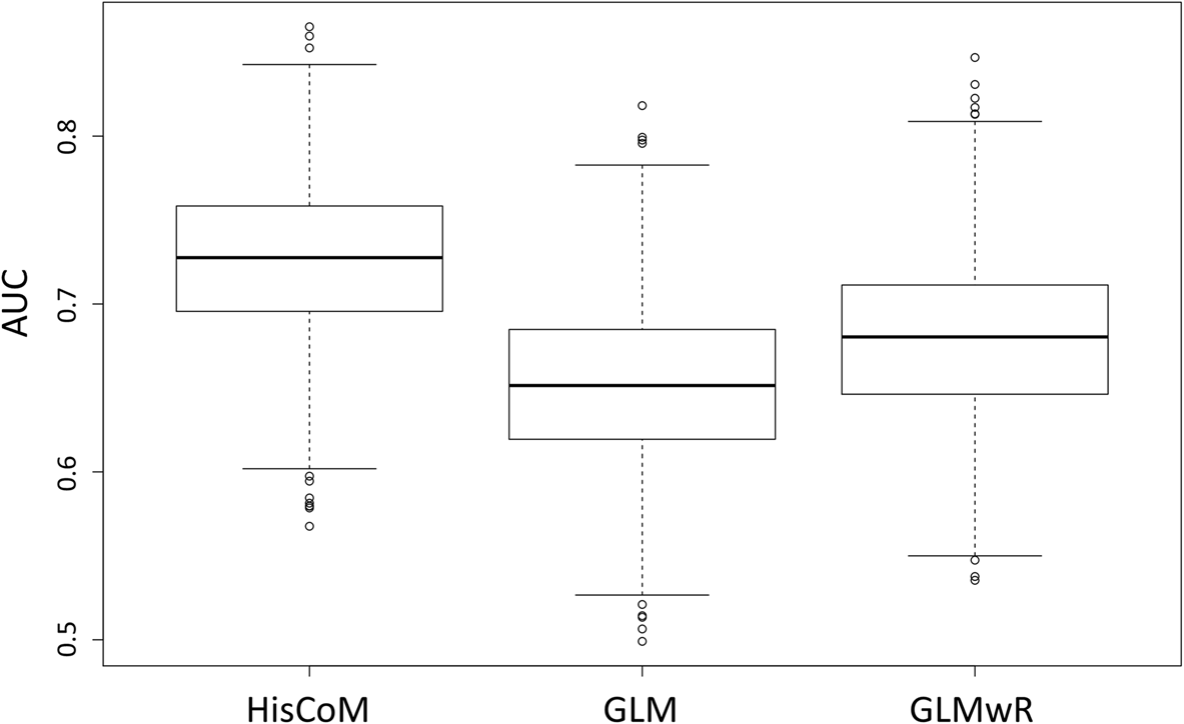
Box plots of ranges of 1000 AUC scores of HisCoM-based simulation data, compared to other models: Simulation model 2

The performance of Simulation model 1 (JCHAIN + RBP4) results, the mean AUC scores of HisCoM, GLM and GLMwR are shown in Table 5. The mean AUC score of HisCoM was 0.8362. Figure 10 shows the range of 1000 AUC scores, as depicted by box plots, with respect to each statistical method. It shows that the HisCoM performed better than others. The performance of Simulation model 2 (RBP4 + APOA1) results, the mean AUC scores of HisCoM, GLM and GLMwR, are shown in Table 6. These results show that HisCoM had the highest mean AUC score, compared to the two other statistical methods. The mean AUC score of model 2 by HisCoM was 0.7270, while the means of the other statistical methods were less than 0.7. Also, Fig. 11 shows the range of 1000 AUC scores, as depicted by box plots, with respect to each statistical method model.

**Table 5:** Mean AUC scores of HisCoM-based Simulation model 1

Methods	Mean AUC
HisCoM	0.8362
GLM	0.8142
GLMwR	0.8018

**Table 6:** Mean AUC scores of HisCoM-based Simulation model 2

Methods	Mean AUC
HisCoM	0.7270
GLM	0.6515
GLMwR	0.6812

In summary, both simulation model results show that HisCoM was the best performing model, compared to the other statistical methods, when there exists a hierarchical structure of MRM-MS data (e.g., peptide to protein).

## Discussion

In this study, we developed a prediction model for tumor response to the multiple tyrosine kinase inhibitor sorafenib (Nexavar®), for liver cancer patients [16], using a component-based structural equation modeling method. We used HisCoM to construct the model, for Korean hepatocellular carcinoma (HCC) patients, using MRM-MS proteomic data, including some demographic variables. HisCoM fit the whole data set at once. In this case, we measured 231 peptides’ weights, and 124 protein’s path coefficients, to the drug response variable, all at once. The positive or negative drug response variables were defined by tumor responses according to mRECIST [20]. Thus, this model can be used for large-scale, structured data, with marker selection (as well as model building), simultaneously. The second, and most important advantage of HisCoM, is that it generates latent variables, which are not directly observed, while collapsing other (observed) variables. For example, our HisCoM combines several collapsed peptides’ MRM-MS data, into several proteins, as latent variables. Unlike other classical methods, such as linear/logistic regression, support vector machine, and random forest, our HisCoM approach considers peptide-to-protein computational structure, and peptide-to-protein biological structure. In the analysis, we found 6 possible protein biomarkers that significantly associate with sorafenib drug response. On the other hand, other classical prediction modeling methods do not consider structure of biological information. Using peptide-level data, we found significant proteins, as possible biomarkers, for building a sorafenib response prediction model for liver cancer patients. The overall work flow, with our statistical analysis, using a HisCoM schema, can be accurately applied not only to other cancers, but also to most any large-scale structured data.

## Conclusions

From possible biomarker selection, to AUC performance test scores, through a model-building process, we compared the performance of our model, constructed using a HisCoM method, to other classical statistical methods such as generalized linear models, using logistic regression (alone) or logistic regression with ridge parameters. For possible drug response biomarkers, 6 significant proteins were statistically selected, using *p*-values, as computed by permutation tests: APOC4 (*p-*value: 0.0061), CD163 (*p-*value: 0.0112), CD5L (*p-*value: 0.0031), JCHAIN (*p-*value: 0.0102), SERPING1 (*p-*value: 0.0142), and RBP4 (*p-*value: 0.0031). All six of these proteins were previously reported as possible biomarkers for hepatocellular cancer (HCC) [31–33]. Of these, CD5L is the best-known HCC biomarker [28]. For the single protein model, using HisCoM, the AUC scores varied from 0.60 to 0.90, depending on the specific protein. For modeling combinations of 2 of the 6 proteins, by HisCoM, the AUC scores varied from 0.73 to 0.95, showing increased performance, compared to single-protein prediction models. On the other hand, AUC scores varied from 0.86 to 0.95, for the 3-protein model, by HisCoM. Finally, using all six of the above-mentioned proteins in the model, we successfully constructed a drug response prediction model using 1-, 2-, 3-, or all six-protein models. By comparing our model’s performance with the generalized linear model with a binary response via ridge penalization, the performance of our six-protein HisCoM prediction model was an AUC score of 0.96, slightly better than the generalized linear model with a binary response via ridge parameter, for the 6-protein panel, with an AUC score of 0.949 (Fig. 8). Thus, both the HisCoM and GLMwR methods had high AUC scores. Overall, we conclude that our model was marginally superior to the classical model types.

For future research, we can apply this overall prediction model-building approach, using HisCoM, to other cancer data especially derived from MRM-MS platform. Since these potential biomarkers were identified in patients’ serum, these could be obtained by a minimally invasive procedure (e.g., as compared to biopsies, lumbar puncture, etc.). Such models could ultimately assist physicians in discerning which therapies might be effective, for individual patients.

## Abbreviations

AUC: Area under the curve
CR: Complete response
GLM: Generalized linear model
GLMwR: Generalized linear model with ridge parameters
GSCA: Generalized structured component analysis
HCC: Hepato cellular carcinoma
HisCoM: Hierarchical structural Component Models
mRECIST: Modified Response Evaluation Criteria in Solid Tumors
MRM-MS: Multiple reaction monitoring mass spectrometry
PD: Progress disease
PHARAOH: Pathway-based approach using HierArchical components of collapsed RAre variants Of High-throughput sequencing data
PR: Partial response
SD: Stable disease
